# Profiling the Tox21 Compound Library for Their Inhibitory Effects on Cytochrome P450 Enzymes

**DOI:** 10.3390/ijms26114976

**Published:** 2025-05-22

**Authors:** Srilatha Sakamuru, Jameson Travers, Carleen Klumpp-Thomas, Ruili Huang, Kristine L. Witt, Stephen S. Ferguson, Steven O. Simmons, David M. Reif, Anton Simeonov, Menghang Xia

**Affiliations:** 1 Division of Pre-Clinical Innovation, National Center for Advancing Translational Sciences, National Institutes of Health, Rockville, MD 20850, USA; sakamurus@mail.nih.gov (S.S.); jameson.travers@nih.gov (J.T.); carleen.klumpp-thomas@nih.gov (C.K.-T.); ruili.huang@nih.gov (R.H.); anton.simeonov@nih.gov (A.S.); 2 Division of Translational Toxicology, National Institute of Environmental Health Sciences, National Institutes of Health, Durham, NC 27709, USAstephen.ferguson@nih.gov (S.S.F.); david.reif@nih.gov (D.M.R.); 3 Center for Computational Toxicology & Exposure, U.S. Environmental Protection Agency, Durham, NC 27711, USA; simmons.steve@epa.gov

**Keywords:** cytochrome P450 (CYP), CYP1A2, CYP2C9, CYP2C19, CYP2D6, CYP3A4, CYP inhibitors, quantitative high-throughput screening (qHTS)

## Abstract

Cytochrome P450 (CYP) enzymes are membrane-bound hemoproteins crucial for drug and xenobiotic metabolism. While more than 50 CYPs have been identified in humans, the isoforms from CYP1, 2, and 3 families contribute to the metabolism of about 80% of clinically approved drugs. To evaluate the effects of environmental chemicals on the activities of these important CYP enzyme families, we screened the Tox21 10K compound library to identify chemicals that inhibit CYP1A2, 2C9, 2C19, 2D6, and 3A4 enzymes. The data obtained from these five screenings were analyzed to reveal the structural classes responsible for inhibiting multiple and/or selective CYPs. Some known structural compound classes exhibiting pan-CYP inhibition, such as azole fungicides, along with established clinical inhibitors of CYPs, including erythromycin and verapamil inhibiting CYP3A4 and paroxetine and terbinafine inhibiting CYP2D6, were all confirmed in the current study. In addition, some selective CYP inhibitors, previously unknown but with potent activity (IC_50_ values < 1 µM), were identified. Examples included yohimbine, an indole alkaloid, and loteprednol, a corticosteroid, which showed inhibitory activity in CYP2D6 and 3A4 assays, respectively. These findings suggest that assessment of a candidate compound’s impact on CYP function may allow pre-emptive mitigation of potential adverse reactions and toxicity during drug development or toxicological characterization of environmental chemicals.

## 1. Introduction

Cytochrome P450s (CYPs) are a large family of enzymes comprising 57 genes in humans. CYPs catalyze the oxidation of numerous hydrophobic chemicals, including endogenous compounds, therapeutic drugs, and environmental toxins [1,2]. Individual CYPs are classified into families and sub-families according to their sequence similarities. CYPs from families 1, 2, and 3 are responsible for the metabolism of 70–80% of clinically used drugs, including CYP1A2, 2A6, 2B6, 2C8, 2C9, 2C19, 2D6, 2E1, and 3A4/5 [1,3]. Drug-metabolizing CYPs are highly expressed in the liver and are also present in other tissues, such as small intestine, lung, kidney, and adipose tissues [4]. In the liver and small intestine, these enzymes are primarily responsible for phase I reactions. Phase I drug metabolism covers a variety of reactions which include oxidation, reduction, and hydrolysis [1]. These reactions introduce polar groups onto the parent compound, converting hydrophobic drugs into more water-soluble compounds facilitating their elimination from the body. CYP gene expression is often regulated by xenobiotic receptors. For example, the aryl hydrocarbon receptor upregulates the expression of CYP1A1 and 1A2 mRNA in response to a range of aromatic hydrocarbon agonists [5]. Furthermore, the constitutive androstane receptor and pregnane X receptor upregulate several xenobiotic-metabolizing enzymes upon activation, including CYP2B6, 2C8, 2C9, 2C19, and 3A4 [6,7]. These CYPs are responsible for metabolizing multiple drugs, leading to altered drug levels and effectiveness. Identifying CYP inhibitors is a critical part of drug development to minimize the potential for drug–drug interactions as required in regulatory guidance from US Food and Drug Administration (FDA) [8] and European Medicines Agency (EMA) [9].

We identified CYP inhibitors from large compound libraries by employing quantitative high-throughput screening (qHTS) using P450-Glo™ assays as a part of the Tox21 program. The Tox21 program aims to assess the potential toxicological effects of a large collection of chemicals (Tox21 10K compound library) using qHTS [10,11]. To represent a more physiologically relevant model in humans, the Tox2110K library has recently been screened using assays that incorporated human liver microsomes to identify chemicals that are either bioactivated or detoxified by metabolism [12,13]. In the present study, five CYP inhibition screens were performed in a qHTS format to obtain datasets consisting of concentration–response curves, potencies, and efficacies for each test compound against human CYP1A2, 2C9, 2C19, 2D6, and 3A4. The compounds are clustered based on their structural similarity and each cluster was evaluated for their enrichment of active (i.e., CYP inhibiting) compounds. We identified several structural classes that inhibit the activity of all five tested CYP enzymes with over 90% of the class members as actives, including azole fungicides, phenylenediamines, and proton pump inhibitors (PPIs). In addition to the pan-active clusters, several CYP-selective clusters were identified, such as polycyclic aromatic hydrocarbons (PAHs) in the CYP1A2 assay. Identifying CYP inhibitors can provide valuable tools for understanding the mechanisms of drug and environmental chemical toxicity.

## 2. Results

### 2.1. qHTS Performances and Reproducibility

**Figure 1:**
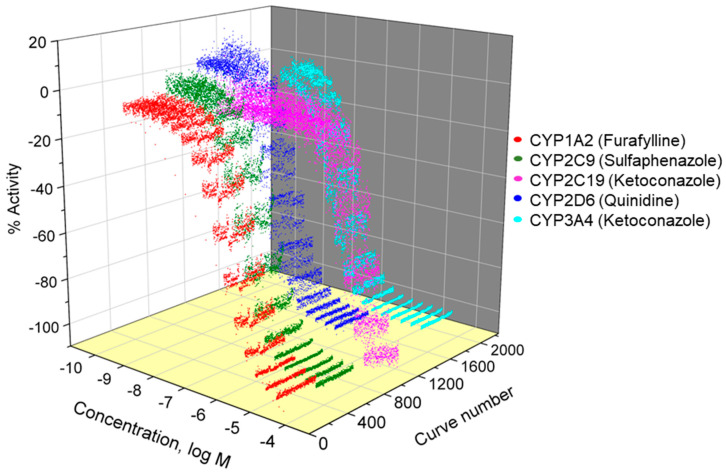
Concentration–response curves of the positive control compounds from five CYP screenings. The positive control compound is plated as 16-point titrations in the control column of every assay plate. The concentration–response curves shown in the figure are from all 408 plates per assay.

The Tox21 10K compound library, consisting of 9667 compounds, was screened in five P450-Glo™ assays, including CYP1A2, 2C9, 2C19, 2D6, and 3A4, to identify CYP inhibitors in a qHTS format. The P450-Glo™ assays utilize a pro-luciferin substrate converted to D-luciferin by specific CYP450 enzymes, with the luminescent signal limited to the amount of converted luciferin providing an indirect measure of CYP450 activity. Loss of signal compared to vehicle-treated controls indicates that a test compound interferes with CYP activity [14]. All five screens performed well in the qHTS format, with performance statistics including signal-to-background (S/B) ratio > 2, coefficient of variation (CV) < 10% and Z′-factor > 0.5, except for CYP2C19 assay, which had a slightly higher CV of 12.3% (Table 1). The positive control compounds, included in all plates as duplicate 16-point titrations, replicated well through the entire screen, as shown in Figure 1, and the IC_50_ values with the standard deviations are given in Table 1.

**Figure 2:**
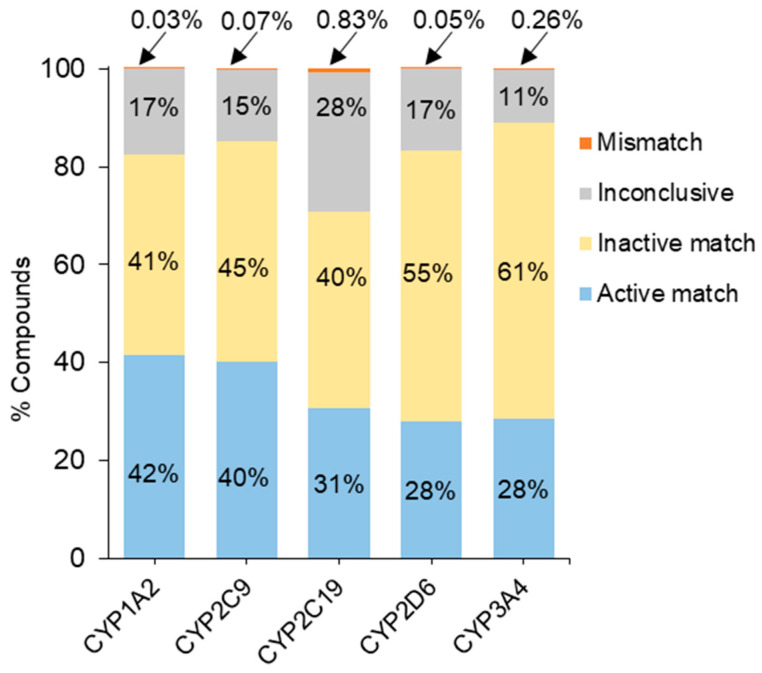
Reproducibility from three runs of five CYP assays. For each assay, the reproducibility was calculated for the 10K compound library with compounds plated in different well locations (three copies) by the active match, inactive match, mismatch, and inconclusive classes.

Based on activities from the three-independent assay runs, compounds were assigned one of the four reproducibility calls: active match, inactive match, inconclusive, and mismatch. All five assays demonstrated good activity reproducibility, with mismatch rates below 1% and active match rates between 28 and 42% (Figure 2).

### 2.2. Identification of Common Structural Clusters from CYP Assays

**Figure 3:**
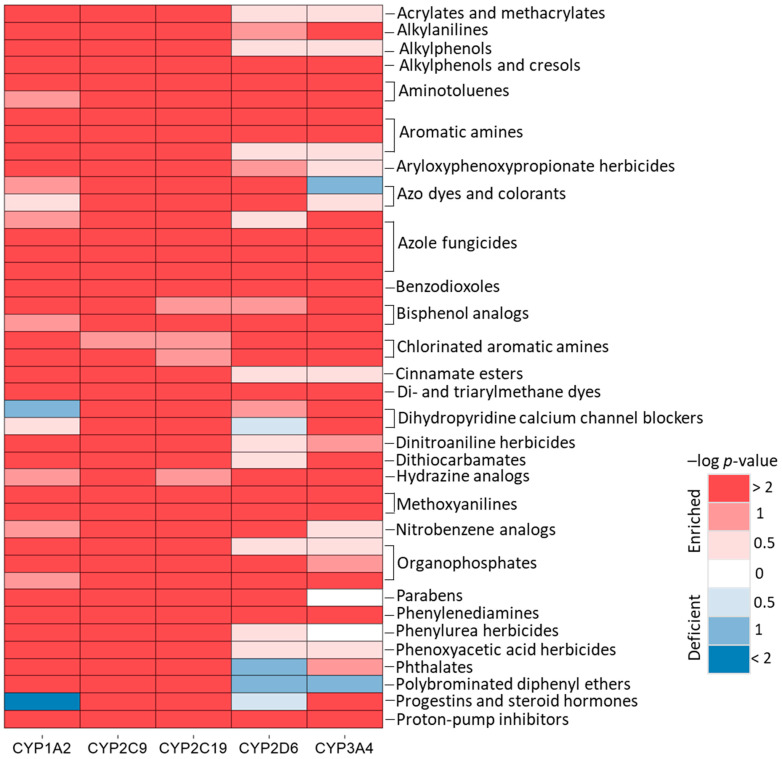
Common structural classes identified from at least three or more CYP assays. The structural classes are enriched with active compounds (inhibitors). The significance of enrichment was determined by *p*-value from Fisher’s exact test. The heat map is colored by the significance (negative logarithmic *p*-value) of enrichment, where the darker shades of red and blue indicates a higher degree of enrichment and deficiency of actives, respectively.

To assess whether structurally similar compounds or compounds sharing the same chemical scaffold have similar CYP inhibition activities, we grouped the Tox21 10K compounds into 1041 clusters based on structural similarity using the Self-Organizing Map (SOM) algorithm [15], such that each cluster represented a structural class. Each cluster was evaluated for its enrichment of the active compounds in each CYP assay. We identified a total of 167 clusters across all five CYPs that are significantly enriched (Fisher’s exact test: *p*-value < 0.01) with CYP inhibitors, each associated with either a specific CYP assay or common among all five CYP assays (Figure S1). The common structural classes identified in three or more CYP assays that are significantly enriched with active compounds are shown in Figure 3. The structural classes, such as azole fungicides, were grouped into three categories, k21.1, k22.1, and k22.2, based on the analogs of different azoles and imidazoles (e.g., econazole, imazalil, and luliconazole) that are known potent CYP inhibitors [16] and were confirmed with IC_50_ values < 2 µM. Proton pump inhibitors (k3.20, e.g., lansoprazole, omeprazole, and pantoprazole) known for their weak inhibition of CYPs in human liver microsomes [17] were confirmed with IC_50_ values of 0.3 µM to 35 µM in all five CYP assays.

We identified that several structural classes composed of chemicals primarily used as herbicides/pesticides are significantly enriched with only CYP1A2, 2C9, and 2C19 inhibitors. These are aryloxyphenoxypropionates (AOPP) (k9.26, e.g., cyhalofop-butyl and fluazifop-butyl); the phenyloxyacetic acid group (k10.26, e.g., cholesolvin, 2,4-D-isobutyl, and clofibrate); the phenylurea group (k26.4, e.g., monolinuron and triclocarbon); and dinitroanilines (k42.11, e.g., isopropalin and benfluralin). Additionally, other classes that are significantly enriched with these three CYP inhibitors include cinnamate esters (k26.23) and polybrominated diphenyl ethers (PBDEs; k28.2). Parabens (k8.15) and phthalates (k13.17) used in personal care products are significantly enriched with all CYP inhibitors except for CYP3A4 and CYP2D6, respectively. Structural classes such as dihydropyridine calcium channel blockers (k10.12, and k11.12) (e.g., nitrendipine, isradipine, and riodipine) and progestins/steroid hormones (k38.25) (e.g., ethisterone, levonorgestrel, and norethindrone) are enriched with CYP2C9, 2C19, and 3A4 inhibitors. Other structural classes of compounds, such as per- and poly-fluoroalkyl substances (PFAS) (k42.12, e.g., lithium perfluorooctane sulfonate) and synthetic opioids/phenyl piperidines (k17.2, e.g., N-(4-Fluorophenyl)-N-[1-(2-phenylethyl)-4-piperidyl] propanamide), were primarily populated with CYP2C9, 2C19, and 2D6 inhibitors. The more potent PFAS compounds and opioids were identified in CYP2C9 [18] and CYP2D6 assays, respectively. The organometallic class (k34.12) was also populated with CYP2C19 and CYP2D6 inhibitors, such as bis(tributyltin)oxide and tributyltin chloride with IC_50_ values < 2 µM.

### 2.3. Identification of CYP Inhibitors

**Figure 4:**
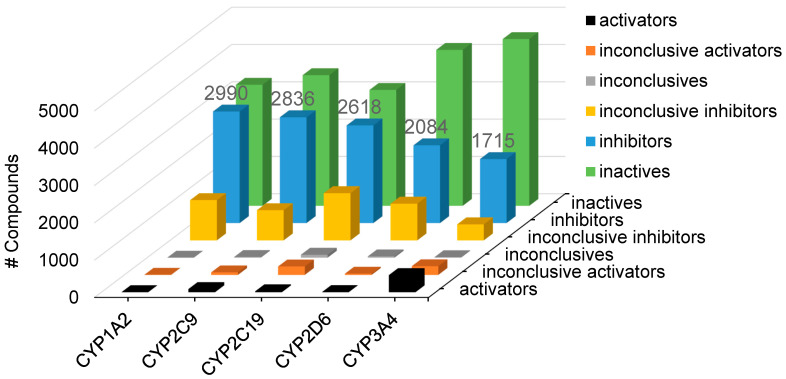
Assay outcomes of the Tox21 10K compounds from five CYP screenings. Each compound was assigned an activity outcome such as activator, inconclusive activator, inconclusive, inhibitor, inconclusive inhibitor, and inactive based on its curve class and efficacy.

All compounds screened against the Tox21 10K compound library in five CYP assays were assigned to one of the following activity outcome categories: activator (compounds exhibiting positive cooperativity), inhibitor, inconclusive activator or inhibitor (due to poor curve quality), inconclusive (activation or inhibition could not be determined among the triplicates), and inactive. The activity distributions for all five CYP screenings are shown in Figure 4. The Tox21 10K compound library consists of 9667 compounds (7316 are unique compounds) that have been screened in all five CYP assays. A total of 4948 (68%) compounds inhibited at least one CYP enzyme and 556 (7.6%) compounds inhibited all five CYP enzymes tested in this study. The number of inhibitors identified for each CYP enzyme ranged from 2990 for the CYP1A2 assay to as few as 1715 for CYP3A4.

**Figure 5:**
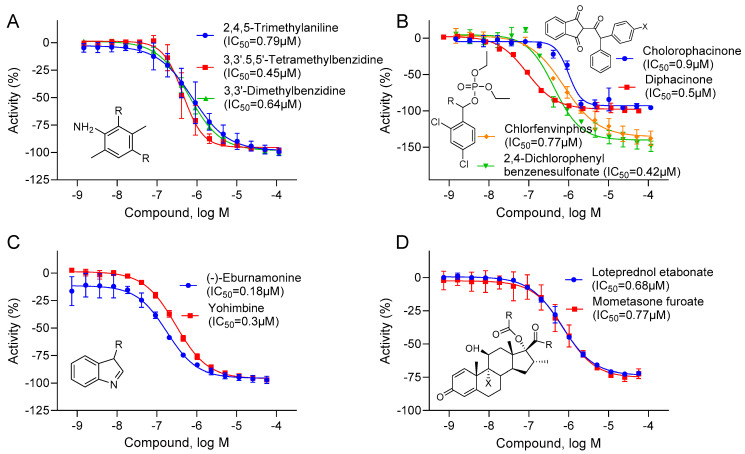
Concentration–response curves of CYP-selective compounds from each CYP assay. (**A**) Arylamines from the CYP1A2 assay; (**B**) indanediones and organophosphates from the CYP2C9 and 2C19 assays, respectively; (**C**) indole alkaloids from CYP2D6 assay; and (**D**) corticosteroids from the CYP3A4 assay. The core structures representing each chemical class are shown in the inserts (with R and X representing alkyl/benzyl groups and halogen, respectively) and the IC_50_ values for each compound are given in parentheses.

The potent active compounds, defined as those with IC_50_ < 1 µM from any one CYP assay, along with their quality control test results of each sample for its identity (confirmed by molecular weight), and purity (>75%) are listed in Table S1. Clusters significantly enriched with inhibitors from specific CYP assays are listed in Table 2, Table 3, Table 4, Table 5 and Table 6. These tables include both structural classes of environmental chemicals and clinical or therapeutic drugs. For example, the structural classes enriched with CYP1A2-specific inhibitors are primarily environmental chemicals, such as chlorinated phenols and anilines used as herbicides, benzimidazoles used as anthelmintics, and phenolic compounds used as disinfectants. In contrast, the structural classes enriched with CYP2D6-specific inhibitors consist mainly of therapeutic drugs, including adrenergic receptor modulators (e.g., beta-blockers), tricyclic antidepressants, and phenothiazines used as antihistamines. The structural classes of compounds (which were less characterized in terms of CYP inhibitors) and potent CYP-selective inhibitors (i.e., with either IC_50_ values < 1 µM or exhibiting potencies differing by greater than 10-fold compared to other CYPs) are shown in Figure 5.

Anthraquinones and PAHs are known to be CYP1A2 inhibitors [19,20], and the clusters consisting of anthraquinones (k19.17, e.g., 2-chloroanthraquinone; and k26.8, e.g., 1,4-diaminoanthraquinone) and PAHs (e.g., 1-nitropyrene in k23.18; 9-bromoanthracene in k27.3; 1-methylpyrene in k28.14; and chrysene in k33.14) were potent CYP1A2 inhibitors in the current study. Several known CYP1A2 inhibitors included in Tox21 10K compound library, such as fluvoxamine (IC_50_ = 0.2 µM ± 0.02), thiabendazole (IC_50_ = 2.1 µM ± 0.4), and zileuton (IC_50_ = 5.3 µM ± 3.3), were all confirmed in the current study. The k28.10 cluster, consisting of aminotoluenes and/or arylamines, was enriched with inhibitors from all five CYP assays, with CYP1A2 exhibiting the most potent inhibitory activities, which were previously unknown for inhibiting CYP1A2 (Figure 5A).

Several clusters were exclusively enriched with inhibitors of enzymes from the CYP2C family, including chloroacetamide/chloroacetanilide herbicides (k18.2, e.g., butachlor), pyrethroid insecticides (k28.23, and k28.24, e.g., resmethrin), antimicrobial/antiseptic agents (k31.1, e.g., dichlorophen), and progestins (k38.26, e.g., deoxycorticosterone). The known CYP2C family inhibitors included in the Tox21 10K compound library, such as amiodarone (IC_50_ = 18.1 µM ± 1.2), fluvastatin (IC_50_ = 3.8 µM ± 1.1), and fluconazole (IC_50_ = 15 µM ± 1.0), were confirmed in the CYP2C9 assay, whereas fluvoxamine (IC_50_ = 0.42 µM ± 0.2), ticlopidine (IC_50_ = 0.41 µM ± 0.2), and ritonavir (IC_50_ = 11 µM ± 1.3) were confirmed in the CYP2C19 assay. The previously unknown and potent CYP2C9 and 2C19 inhibitors that belong to indanedione (k18.15) and organophosphate (k26.1) classes are shown in Figure 5B.

The highest number of CYP-selective clusters enriched with actives were from CYP2D6 (Table 5). The CYP2D6 enzyme is responsible for metabolizing many commonly prescribed drug classes, including antiarrhythmics, antidepressants, antipsychotics, beta-blockers, synthetic opioids, and tricyclic antidepressants [21]. The representative chemicals from these drug classes that were included in the Tox21 10K compound library, such as flecainide (IC_50_ = 1.7 µM ± 0.7), fluoxetine (IC_50_ = 0.5 µM ± 0.2), chlorpromazine (IC_50_ = 0.3 µM ± 0.03), labetalol (IC_50_ = 0.6 µM ± 0.2), norfentanyl (IC_50_ = 4.5 µM ± 0.6), and trimipramine (IC_50_ = 3.1 µM ± 0.01), and other potent CYP2D6 inhibitors, such as paroxetine (IC_50_ = 0.7 µM ± 0.07) and terbinafine (IC_50_ = 0.4 µM ± 0.11), were all confirmed in the current study. The k40.4 cluster, consisting of indole alkaloids, was enriched with CYP2D6 inhibitors that were previously unknown to inhibit CYP2D6, such as eburnamonine and yohimbine (Figure 5C).

CYP3A4 is responsible for the metabolism of more than 50% of commonly prescribed drugs, and some of the known CYP3A4 inhibitors belong to the anti-bacterial, anticancer, anti-viral, and antihypertensive classes [22]. Representative chemicals from these classes in the Tox21 10K compound library, including erythromycin (IC_50_ = 0.3 µM ± 0.04), tamoxifen (IC_50_ = 5.8 µM ± 1.0), ritonavir (IC_50_ = 0.03 µM ± 0.01), and verapamil (IC_50_ = 0.9 µM ± 0.5), were all confirmed as CYP3A4 inhibitors. The clusters consisting of corticosteroids (k42.25, k42.26) in the Tox21 10K compound library were highly enriched with potent CYP3A4 inhibitors that were previously unknown to inhibit CYP3A4, such as mometasone furoate and loteprednol etabonate (Figure 5D).

### 2.4. Identification of Luciferase Inhibitors

To exclude potential interference with the luciferase signal, the Tox21 10K compounds were screened in a luciferase assay. The results showed that 9% of the compounds exhibited luciferase inhibitory activity, 84% were inactive, and the remaining 7% yielded inconclusive results. The luciferase activity data for the potent (IC_50_ < 1 µM) CYP inhibitors are summarized in Table S1, where most compounds were shown to be either inactive or less potent (IC_50_ > 20 µM) in the luciferase assay.

### 2.5. Identification of CYP Activators

In addition to CYP inhibition, some compounds also demonstrated CYP activation due to the allosteric effect on the probe substrate reaction in these assay systems [23]. The highest number of activators were identified from the CYP3A4 assay, followed by the 2C9 and 2C19 assays. The activators identified in each CYP assay are listed in Table S2. The structural classes used as herbicides/pesticides are particularly populated with CYP3A4 activators. They include AOPP (e.g., quizalofop-ethyl in k9.26); the phenyloxyacetic acid group (e.g., 2,4-D 1-butyl ester in k10.26); chlorinated or brominated phenols (e.g., 2,4,5-trichlorophenol in k29.1); nitriles (e.g., bromoxynil in k29.2); nitrophenyl ether (e.g., nitrofen in k29.3); and organophosphates (e.g., cresyl diphenyl phosphate in k29.14). Other structural classes populated with CYP3A4 activators include benzoate esters (e.g., 2-phenylethyl benzoate in k12.16) and steroids (e.g., drospirenone in k36.26, guggulsterone E in k37.26, and megestrol in k38.26).

## 3. Discussion

The Tox21 10K compound library, consisting of 7316 unique compounds, was screened in five CYP assays for human CYP1A2, 2C9, 2C19, 2D6, and 3A4 using P450-Glo™ assay technology [14,24]. Different luciferin substrates were used for each P450-Glo™ assay, as these substrates are specific to each CYP enzyme [25]. The five CYP screenings performed in three independent runs exhibited high reproducibility, with minimal activity mismatch rates. Of the 7316 compounds tested, 68% were active inhibitors in at least one CYP assay and 7.6% demonstrated inhibitory activity against all five CYP enzymes. Notably, CYP2C9 and 2C19 have the most overlapping active compounds, likely related to their high structural and functional similarity among the five CYPs included. The Tox21 10K compounds were clustered based on structural similarity, and each structural cluster was evaluated for its enrichment of active inhibitors in five CYP assays. Structural analysis has identified both pan-CYP and/or CYP-selective clusters that are enriched with actives, such as azole fungicides, proton pump inhibitors, and/or PAHs, respectively [5,16,17] that were confirmed in the current study. Previously unknown potent compounds (IC_50_ values less than 1 µM) were identified from the arylamine, indanedione, organophosphate, indole alkaloid, and corticosteroid classes in CYP1A2, 2C9, 2C19, 2D6, and 3A4 assays, respectively. These potentially novel compounds were either inactive or less potent (IC_50_ > 20 µM) in the luciferase counter-screen assay, indicating minimal interference with the luciferase signal. Although the inhibitory potential of these compounds has yet to be validated in vivo, several publicly or commercially available tools, such as ADMET-AI [26] or ADMET Predictor^®^ from Stimulation Plus, can be used to rapidly and accurately predict their ADMET properties, a critical step for identifying drug-like candidates. Some therapeutic compounds from the aforementioned chemical classes, such as yohimbine and loteprednol etaboanate, have Cmax (maximum plasma concentration) values of 75 ng/mL and 139 pg/mL, respectively [27,28,29]. The IC_50_ values for these drugs in the current study are 0.3 µM and 0.68 µM, respectively. For yohimbine, the ratio of IC_50_ and Cmax value is less than 10, suggesting it may be an effective inhibitor within the therapeutic range [30]. In contrast, the IC_50_ value for loteprednol in this study may not fall within the comparable therapeutic range, particularly for drugs primarily administered topically or intranasally [28,29].

Surprisingly, activators for CYP2C9, 2C19, or 3A4 were also identified during this study. However, the apparent activators identified in biochemical-based in vitro assays are not true endpoints of gene induction, which entails the activation of CYP gene expression. For example, common CYP inducers, including carbamazepine, phenobarbital, phenytoin, and rifampicin [31], were all shown to be either inactive or inhibitors in the current study. This apparent activation of CYPs indicates positive cooperativity, in which the binding of a substrate to the active site increases the affinity of other binding sites [32]. This phenomenon, particularly associated with the CYP3A4 enzyme, has been reported as heterotropic cooperativity [33], which involves the simultaneous binding of two different substrates to the spatially distinct substrate-binding domains within the enzyme active site or effector site [34] and hence results in the formation of distinct enzyme–substrate complexes. Although the detailed mechanism underlying allosteric or heterotropic cooperativity in CYP3A4 activation remains unclear, in silico approaches may offer valuable tools for evaluating these interactions. Previous studies have identified potential ligand-binding sites in CYP3A4 and highlighted Phe123 as a key residue mediating heterotropic allosteric interactions [35,36].

The five CYP enzymes included in the current study, representing the CYP 1, 2, and 3 families, are responsible for the biotransformation of 70–80% of clinically approved drugs [1,3]. Several drugs and environmental chemicals act as both substrates and inducers or inhibitors of CYPs. This is due to non-selectivity, i.e., the same drug can act as a substrate for several CYP isoforms, leading to the formation of reactive or secondary metabolites that can inhibit or induce CYPs [37]. Moreover, the assay technology used in the current study (P450-Glo™) is unable to distinguish between inhibitors and substrates, and additional studies such as metabolic stability assays are needed to make those distinctions. As previously reported [38], the substrate depletion method can be used to determine the intrinsic clearance of the compound and evaluate its metabolic stability. Drug classes such as beta-blockers and corticosteroids, identified as inhibitors in this study, are known to be extensively metabolized by CYP2D6 and 3A4, respectively [21,39]. However, the structural classes included in the current study are populated with selective inhibitors identified in the corresponding CYP assays. The CYP2D6 and 3A4 assays demonstrated the highest activity across several classes of therapeutic drugs, whereas CYP2C19 had the lowest activity rate within these therapeutic drug classes, which is not surprising given that CYP2D6 and 3A4 contribute to over 50% of CYP-related drug metabolism [1].

Among the five CYPs, CYP3A4 is notable for exhibiting substrate-dependent inhibition due to its multiple substrate’s sites. The three distinct substrate recognition sites are typically grouped based on representative substrates, testosterone, midazolam, and nifedipine, with most other substrates falling within one of these categories [40]. The probe substrate used in the current study, luciferin-PPXE [25], is known to be inhibited by midazolam and nifedipine. In the current study, both compounds demonstrated CYP3A4 inhibition, with IC_50_ values of 11.4 µM for midazolam and 13.4 µM for nifedipine. Therefore, any compound that inhibits a CYP3A4 assay using either of those substrates is also expected to inhibit the luciferin-PPXE assay. In contrast, testosterone has been shown to stimulate the luciferin-PPXE reaction. In the current study, testosterone activated CYP3A4 with an AC_50_ of 4.2 µM, suggesting that compounds binding in a similar manner may also enhance the assay signal. Thus, the luciferin-PPXE assay can detect effects from compounds interacting with any of the three known substrate-binding configurations. Importantly, luciferin-PPXE is the only DMSO-tolerant probe substrate for CYP3A4, which is critical for screening purposes, as all test compounds in this study were dissolved in DMSO, resulting in a final concentration of 0.5% DMSO in the assay.

The screening data revealed that most structural classes of compounds exhibited non-selective CYP inhibition due to the high homology in the binding pocket regions of the CYPs, all of which contain the heme iron motif. The majority of the potent inhibitors identified across all CYP assays are azole compounds, suggesting that their potency arises from the presence of heme-binding groups in their chemical structures [41]. Several chemical classes, including those from the PAHs and quaternary ammonium compounds (QACs) found throughout the library, were enriched with CYP1A2 and 2D6 inhibitors, respectively, as most of these chemicals inhibit CYPs through a mechanism-based action. This process leads to the formation of reactive metabolites that covalently bind to the enzymes resulting in their inactivation [42]. However, CYP-selective inhibitors pose drug interaction challenges, particularly when targeting enzymes with high homology. In addition to enzyme inactivation, reactive metabolites can covalently bind to other cellular macromolecules leading to adverse effects such as hepatotoxicity and carcinogenicity. This also includes the metabolic activation of specific natural products from dietary supplements that can result in toxicities [43,44]. Studies have shown that a polyphenol-rich diet from plant-based foods, along with parabens and phthalates used in consumer products, can inhibit expression of some CYP isoforms [45,46]. Therefore, evaluating the potential of a drug candidate or an environmental chemical to inhibit CYP activity is a crucial step in therapeutic drug development or chemical synthesis.

The current study identified potential CYP inhibitors from the Tox21 10K compound library and assessed their CYP inhibitory activities by clustering them based on structural similarity, resulting in the identification of several pan-CYP and CYP-selective chemical classes. These screening datasets offer valuable insights into the chemical classes associated with CYP inhibition, which could significantly aid early-stage drug development and chemical synthesis. Additionally, given that the Tox21 10K library contains more than two-thirds environmental chemicals, these data may also be instrumental in predicting toxicity from the biotransformation of these chemicals.

## 4. Materials and Methods

### 4.1. Reagents and Compounds

The P450-Glo™ assay systems (Promega Corp., Madison, WI, USA) were used for the screenings to identify the compounds that inhibit CYP1A2, 2C9, 2C19, 2D6, and 3A4 activities. Positive control compounds were purchased from Sigma-Aldrich (St. Louis, MO, USA): furafylline (CASRN 80288-49-9) for CYP1A2, sulfaphenazole (CASRN 526-08-9) for CYP2C9, quinidine (CASRN 56-54-2) for CYP2D6, and ketoconazole (CASRN 65277-42-1) for both CYP2C19 and 3A4 assays. PTC124 (CASRN 775304-57-9) used as a positive control for luciferase assay was purchased from Santa Cruz Biotechnology, Inc. (Dallas, TX, USA).

### 4.2. qHTS of CYP Assays

The components of the enzyme–substrate mixture are provided in Table 7. The CYP assays were performed as previously described [47]. The enzyme–substrate mixture supplemented with 0.1% Bovine Albumin Fraction V (ThermoFisher Scientific Inc., Waltham, MA, USA) was dispensed into each well (2 µL per well) of white opaque 1536-well plates (Greiner Bio-One North America Inc., Monroe, NC, USA) using a BioRAPTR Flying Reagent Dispenser (FRD, Beckman Coulter, Brea, CA, USA). Positive control and test compounds dissolved in DMSO were transferred to the assay plates at 23 nL using a Pintool station (Wako, San Diego, CA, USA) to columns 1–4 and 5–48 of the assay plates, respectively. After compound addition, the assay plates were incubated at ambient temperature (20–22 °C) for 10 min. Next, 2 µL of NADPH regeneration solution was added to each well using the FRD. The assay plates were incubated for 1 h at an ambient temperature for the CYP1A2, 2D6, and 3A4 assays or at 37 °C for the CYP2C9 and 2C19 assays. The reaction was stopped by adding 4 µL of luciferase detection reagent using an FRD and incubating at ambient temperature for an additional 20 min. Luminescent signal was measured using a ViewLux plate reader (PerkinElmer, Shelton, CT, USA). Raw data were expressed as relative light units (RLU). The Tox21 10K test compounds were screened at 15 concentrations ranging from 0.7 nM to 58 µM in three independent experiments. A schematic outlining the assay workflow is presented in Figure S2.

### 4.3. Luciferase Assay

A total of 3 µL of substrate mix containing 10 µM each of D-luciferin and ATP was dispensed in white opaque 1536-well plates using an FRD. Positive control (PTC124) and test compounds dissolved in DMSO were transferred to the assay plates at 23 nL using a Pintool station to columns 1–4 and 5–48 of the assay plates, respectively. After compound addition, 1 µL of 10 nM *Photinus pyralis* (firefly) luciferase was added using an FRD. Luminescent signal was measured using a ViewLux plate reader. Raw data were expressed as RLU. The Tox21 10K compounds were screened at 15 concentrations from 0.7 nM to 58 µM in three independent experiments.

### 4.4. qHTS Data Analysis

The qHTS data were analyzed according to the previously described protocol [48]. Briefly, the raw data were normalized at the assay plate level from 0% using the positive control wells for each CYP assay and 100% using DMSO-only wells, as follows: % Inhibition = [(V_compound_ − V_DMSO_)/(V_DMSO_ − V_positive control_)] ∗ 100, where V_compound_ represents the compound RLU, V_positive control_ and V_DMSO_ represents the median RLU of the positive control and DMSO-only wells, respectively. An internal pattern correction algorithm was applied using assay plates treated with DMSO only, placed at the beginning of the compound plate stack to identify any inconsistent background patterns unrelated to test compound treatment. Corrected plate data were pivoted to form concentration–response series, which were subsequently fit to a four-parameter Hill equation yielding half-maximal inhibitory concentration (IC_50_) and maximal response (efficacy) values. Compounds were designated as Curve Classes 1–4 according to the type of concentration–response curve obtained based on efficacy, the number of data points observed above the background activity, and the quality of fit. Each curve class combined with an efficacy cutoff was further converted to a numerical curve rank according to previously described criteria [48]. Curve ranks from triplicate runs of each compound were then averaged. Based on the average curve rank from the triplicate runs and the reproducibility call, each compound was assigned an activity outcome, such as an inhibitor, inconclusive inhibitor/activator (due to poor curve quality), activator, inconclusive (activity direction could not be determined), or inactive.

The performance of each assay was also assessed by measuring reproducibility of the three independent assay runs screened against three Tox21 10K compound library copies, with compounds plated in different well locations within each copy. Reproducibility of triplicate runs was calculated, as previously described [48]. Each concentration–response curve was first assigned an activity outcome based on the curve classes as follows: active (classes −1.1, −1.2, −2.1, or −2.2), inactive (class 4), and inconclusive (all remaining classes). Each activity outcome category was then assigned a score. The pair-wise activity outcome score differences for all replicate curves of each compound were then averaged. The percentage of inactive calls for the compounds was calculated to determine the final reproducibility call such as, active match (average score difference < 1.1, % inactive call < 25%), inactive match (average score difference < 1.1, % inactive call > 50%), mismatch (average score difference > 2.5), and inconclusive (all other cases).

The Tox21 10K compounds were grouped into 1041 clusters based on structural similarity (Leadscope^®^ fingerprints: Leadscope Inc., Columbus, OH, USA) using the self-organizing map (SOM) algorithm [15]. Each cluster was evaluated for its enrichment of active compounds with respect to a CYP. For each CYP, the fraction of actives in each cluster was determined and compared to the library average fraction of actives, and a statistical significance of enrichment was determined by Fisher’s exact test (*p*-value < 0.01). Each cluster consisting of structurally similar compounds is represented as a hexagon as shown in Figure S1, and hexagons are colored based on their negative logarithmic scale of the *p*-value. Neighboring clusters contain more similar structures than distant ones, and each cluster (hexagon) in the current study was identified by the row number followed by the column number, such as the cluster ID “k42.8” represents the hexagon in row 42, and column 8 (Figure S1).

## Figures and Tables

**Table 1:** Screening statistics of the CYP assays.

Assay	S/B	CV (%)	Z′-Factor	IC_50_ (µM) (Positive Control)
CYP1A2	20.9 ± 5.0	2.0 ± 0.9	0.92 ± 0.03	1.21 ± 0.16 (Furafylline)
CYP2C9	21.3 ± 1.6	3.2 ± 0.9	0.87 ± 0.03	0.25 ± 0.03 (Sulfaphenazole)
CYP2C19	9.4 ± 2.3	12.3 ± 2.8	0.50 ± 0.10	21.85 ± 4.24 (Ketoconazole)
CYP2D6	17.6 ± 2.6	7.0 ± 1.8	0.76 ± 0.06	0.01 ± 0.002 (Quinidine)
CYP3A4	22.42 ± 1.9	2.4 ± 0.9	0.91 ± 0.03	0.06 ± 0.01 (Ketoconazole)

Data represent mean ± standard deviation. S/B = signal-to-background ratio; CV = coefficient of variation; IC_50_ = half-maximal inhibitory concentration.

**Table 2:** Representative structural classes enriched with CYP1A2-selective inhibitors.

Cluster #	Structural Class	Description	Example
k7.9	Benzimidazoles	Anthelmintics	Parbendazole
k7.10	Benzimidazoles	Anthelmintics	Cyclobendazole
k10.14	Anthranilic acids	Fragrance agents	Phenethyl anthranilate
k10.18	Phenolic compounds	Disinfectants	Guaiacol carbonate
k12.16	Benzoates	Various applications	Hexane-1,6-diyl dibenzoate
k16.7	Acetanilide	Analgesic	Phenacetin
k19.17	Anthraquinones	Dyes, Pigments	2-Chloroanthraquinone
k21.18	Nitrobenzaldehydes	Pharmaceutical agents	4-Chloro-3-nitrobenzaldehyde
k25.13	Alkyl phenols	Various applications	3,5-Dimethylphenol
k27.3	Aryl bromides	Fire-retardants	Hexabromobenzene
k27.9	Nitroanilines	Pharmaceutical agents	2-Nitro-4-thiocyanoaniline
k27.23	Benzyloxycarbonyls	Polymer agents	Benzyl methacrylate
k29.5	Chlorinated anilines	Herbicides	2,4-Dichloro-6-nitroaniline
k29.23	Esters	Flavoring agents	Dimethyl succinylsuccinate
k30.2	Chlorinated phenols	Disinfectants	6-Chlorothymol
k31.2	Chlorinated phenols	Insecticides	2-Chloro-4-phenylphenol
k32.5	Triazines	Herbicides	Cybutryne
k33.14	Polycyclic aromatic hydrocarbons (PAHs)	Carcinogens	Benzo(e)pyrene
k42.13	Pyrroles/pyrazoles/pyridines	Insecticide	Chlorfenapyr

**Table 3:** Representative structural classes enriched with CYP2C9-selective inhibitors.

Cluster #	Structural Class	Description	Example
k1.12	Flavonolignans	Antioxidants	Silybin
k4.14	Benzophenones	Sunscreen agents	Mexenone
k11.25	Phenoxy acetic acids	Herbicides	2,4-DB-isooctyl ester
k11.26	Phenoxy acetic/propionic acids	Herbicides	Dichlorprop
k13.23	Indole acetic acids	Nonsteroidal anti-inflammatory drugs	Proglumetacin
k20.3	Benzodiazepines	Cholecystokinin antagonists	Devazepide
k20.18	Diphenyl ethers	Herbicides	Oxyfluorfen
k24.2	Chlorobenzenes	Pesticides	Hexythiazox
k24.15	Nitrobenzene sulfonates	Dyes	Disodium 4,4′-dinitro-2,2′-stilbenedisulfonate
k24.20	Vitamin K antagonists	Anticoagulants	Dicumarol
k25.8	Sulfonic acid analogs	Dyes, pigments	2-Amino-1-naphthalenesulfonic acid
k26.13	Sulfonylurea analogs	Anti-diabetic medication	Glyoctamide
k29.2	Brominated phenols	Flame retardants, herbicides	3,3′,5,5′-Tetrabromobisphenol A
k29.3	Phenolic compounds	Anti-bacterial agents, herbicides	Triclosan
k33.20	Aldehydes	Various applications	Crotonaldehyde

**Table 4:** Representative structural classes enriched with CYP2C19-selective inhibitors.

Cluster #	Structural Class	Description	Example
k11.19	Cinnamates, strobilurins, retinoids	Various applications	Picoxystrobin
k23.10	PAHs, dyes	Various applications	9-Phenanthrol
k26.1	Chlorinated compounds	Pesticides	Dichlofenthion
k28.5	Anilines	Precursors of azo dyes	4-Bromoaniline
k32.20	Aldehydes	Various applications	Phenylacetaldehyde
k35.20	Carbamates	Pesticides	Terbucarb

**Table 5:** Representative structural classes enriched with CYP2D6-selective inhibitors.

Cluster #	Structural Class	Description	Example
k2.18	Monocarboxylic acid amides	Therapeutic drugs	Flecainide
k4.5	Adrenergic receptor modulators	Beta-blockers, bronchodilators	Pronethalol
k5.13	Benzophenones	UV absorbers in cosmetics	2,2′,4,4′-Tetrahydroxybenzophenone
k6.26	Amines, thiazolidinediones, and Selective Estrogen Receptor Modulators	Therapeutic drugs	Bazedoxifene
k9.21	Sulfite esters, tetrazines	Acaricides	Propargite
k23.1	Pyrimidines, monochlorobenzenes	Acaricides, fungicides	Fenarimol
k24.4	Biguanides and other chemicals	Disinfectants	Chlorhexidine
k27.17	Imidazolium-based ionic liquids	Organic solvents	1-Hexadecyl-3-methylimidazolium chloride
k28.16	Quaternary ammonium compounds (QACs)	Disinfectants, antiseptics, and surfactants	Cetylpyridinium bromide
k30.14	Organo-metallic compounds	Herbicides, fungicides	Phenylmercuric acetate
k31.8	Aminopyridines	Acetylcholinesterase inhibitors	Ipidacrine
k32.10	Amines	Surfactants	(Z)-9-Octadecenylamine
k34.9	Ethylenediamines	Solvents	N-Ethyl-N-(3-methylphenyl) ethane-1,2-diamine
k36.14	QACs	Disinfectants, antiseptics	Ethylhexadecyl dimethylammonium bromide
k36.15	QACs	Disinfectants, antiseptics	Benzylhexadecyl dimethylammonium chloride
k37.9	Indanes and other chemicals	Class I antiarrhythmic and therapeutic drugs	Aprindine hydrochloride
k38.9	Pyrrolidin-2-ones and Morpholines	Respiratory stimulants, antidepressants	Doxapram hydrochloride
k39.2	5-HT2A receptor antagonists	Antipsychotic drugs	Spiperone
k39.3	Benzimidazoles, heteroarylpiperidines	Neuropsychiatric drugs	Domperidone
k39.5	Ergot alkaloids	Psychoactive drugs	Metergoline
k39.8	2-benzylaminopyridines	Antihistamines	Chloropyramine hydrochloride
k39.9	Ethanolamines	Antihistamines	Diphenhydramine hydrochloride
k40.2	Piperidines, aromatic ketones	Antispasmodics	Tolperisone hydrochloride
k41.8	Tricyclic antidepressants	Depressive disorder therapeutics	Clomipramine hydrochloride
k42.2	Anticholinergics/antimuscarinics	Therapeutic drugs	Pipethanate ethylbromide
k42.5	Phenothiazine	Antihistamines	Trimeprazine tartrate

**Table 6:** Representative structural classes enriched with CYP3A4-selective inhibitors.

Cluster #	Structural Class	Description	Example
k1.10	Anthracyclines	Chemotherapeutic drugs	Nemorubicin
k1.13	Flavonoids	Phytochemicals	Biochanin A
k2.9	Ansamycins	Antimicrobials	Rifampicin
k2.16	Sulfonyl urea	Anti-diabetic drugs	Glybenclamide
k7.15	Resorcinols	Macrolides	Zearalenone
k9.7	Vitamin B1 analogs	Supplements	Dicethiamine hydrochloride
k12.14	Carbonylimidazoles	Anesthetics, antimicrobials	Propoxate
k23.2	Benzimidazoles	Antifungal drugs	Clotrimazole
k23.11	Phenols, polyphenols	Topical dermatological medication	4-Hexylresorcinol
k23.20	Furocoumarin	Phytochemical, anti-inflammatory	Imperatorin
k28.8	Sulfonamides	Anti-bacterial drugs	Dapsone
k42.25	Corticosteroids	Anti-inflammatory drugs	Budesonide
k42.26	Corticosteroids	Anti-inflammatory drugs	Loteprednol etabonate

**Table 7:** Reaction components for the enzyme–substrate mixture in the P450-Glo™ assays.

Assay	CYP Enzyme	KPO_4_ Concentration	Substrate Concentration	Incubation Time
CYP1A2	0.01 pmol/µL	100 mM	100 µM Luciferin-ME	60 min (RT)
CYP2C9	0.01 pmol/µL	25 mM	100 µM Luciferin-H	60 min (37 °C)
CYP2C19	0.005 pmol/µL	50 mM	10 µM Luciferin-H EGE	60 min (37 °C)
CYP2D6	0.005 pmol/µL	100 mM	30 µM Luciferin-ME EGE	60 min (RT)
CYP3A4	0.01 pmol/µL	100 mM	25 µM Luciferin-PPXE	60 min (RT)

RT = room temperature.

## Data Availability

The screening data are available at https://tripod.nih.gov/pubdata/ (accessed on 7 April 2025) and in PubChem at https://pubchem.ncbi.nlm.nih.gov/ (accessed on 7 April 2025) (PubChem Assay IDs for CYP assays are 1671199, 1671198, 1671197, 1671196, and 1671201 and for Luciferase assay is 1224835).

## Supplementary Materials

The supporting information can be downloaded at https://www.mdpi.com/article/10.3390/ijms26114976/s1.

## Abbreviations

CYP	Cytochrome P450
qHTS	Quantitative high throughput screening
PPI	Proton pump inhibitor
PAH	Polycyclic aromatic hydrocarbon
RLU	Relative light units
SOM	Self-organizing map
S/B	Signal-to-background ratio
CV	Coefficient of variation
AOPP	Aryloxy phenoxy propionate
TCA	Tricyclic antidepressant
QAC	Quaternary ammonium compound
