# Supplementary material for: Profiling the Tox21 Compound Library for Their Inhibitory Effects on Cytochrome P450 Enzymes
Source: Int J Mol Sci. 2025 May 22;26(11):4976. doi: 10.3390/ijms26114976 (PMC12155096; doi:10.3390/ijms26114976)

Figure S1. Structure-activity relationship analysis of the Tox21 10K compounds. Each hexagon represents a cluster of structurally similar compounds. The color gradient (negative logarithmic *p*-values) is indicative of the enrichment of CYP actives in that specific cluster. The maroon/dark red hexagons are enriched with actives and dark blue hexagons are deficient of actives. The white hexagons represent the fraction of active compounds in that cluster, which is close to the library average. Gray hexagons represent clusters with no compounds.

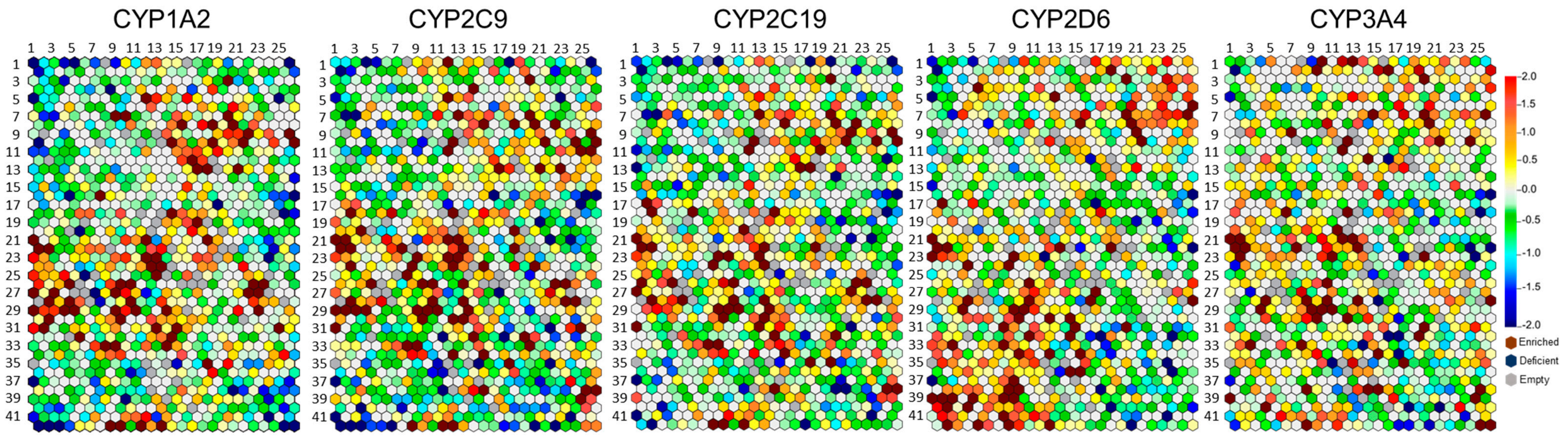

Figure S2. Work flow of the P450-Glo™ assays.

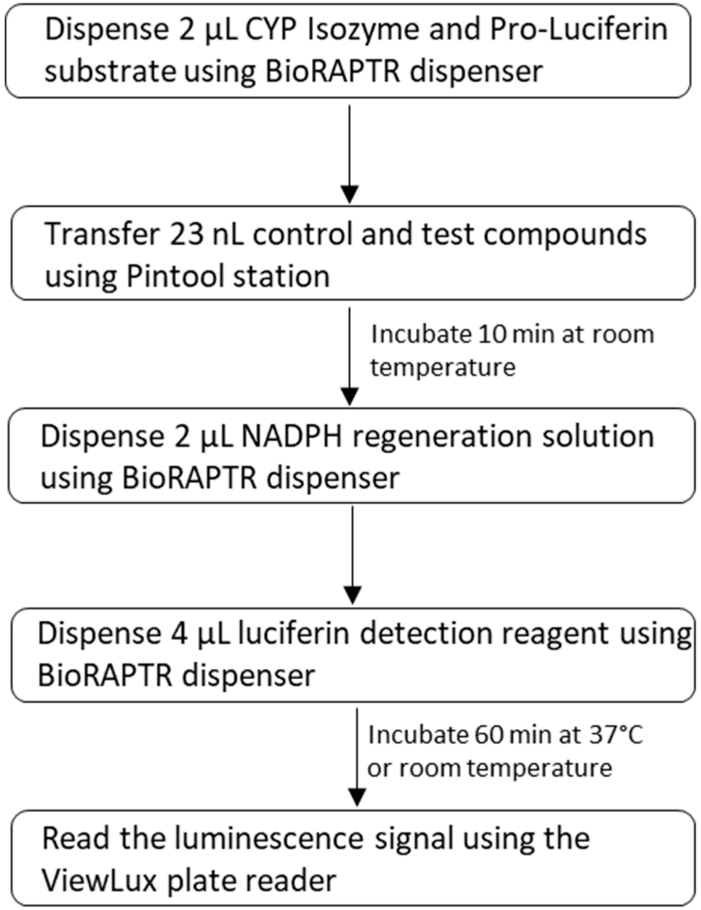

Supplement: Supplementary file 1 [file ijms-26-04976-s001.zip › Supplemental Material.pdf]
